# 早期非小细胞肺癌淋巴结转移规律与清扫方式研究进展

**DOI:** 10.3779/j.issn.1009-3419.2016.06.12

**Published:** 2016-06-20

**Authors:** 宁宁 丁, 友生 毛

**Affiliations:** 100021 北京，国家癌症中心，协和医学院，中国医学科学院肿瘤医院胸外科 Department of Thoracic Surgery, Chinese Academy of Medical Sciences Cancer Hospital, Peking Union Medical College, National Cancer Institute, Beijing 100021, China

**Keywords:** 肺肿瘤, 淋巴结转移, 纵隔淋巴结清扫, 纵隔淋巴结取样, Lung neoplasms, Lymph node metastasis, Mediastinal lymph node dissection, Mediastinal lymph node sampling

## Abstract

目前，肺癌已是全球范围内发病率及死亡率最高的恶性肿瘤，非小细胞肺癌（non-small cell lung cancer, NSCLC）约占肺癌80%。手术治疗在早期NSCLC治疗中占主导地位，而淋巴结分期及手术中清扫程度直接影响着患者术后生活质量及患者的预后。解剖性肺叶切除加系统性淋巴结清扫一直以来被认为是NSCLC的标准手术方式，但对早期NSCLC患者纵隔淋巴结清扫程度问题上一直存在较大争议，精确评估区域淋巴结的转移及淋巴结清扫的程度是影响患者围手术期并发症和预后的重要因素。对于早期肺癌行肺叶特异性或选择性淋巴结清扫已逐渐为国内外学者接受，并可能成为临床Ⅰ期NSCLC患者标准淋巴结清扫方式。

## Abstract (en)

Lung cancer ranks the first position in morbidity and mortality among all malignances in China. Non-small cell lung cancer (NSCLC) accounts for nearly 80% of all lung malignancies. Surgical resection is still the current major treatment method for early stage NSCLC. Lymph node stages together with the extent of lymph node dissection directly affect the prognosis. Anatomical lobectomy with systematic mediastinal lymph node dissection have been the standard surgical treatment for NSCLC. However, it is controversial in the extent of lymph node dissection for early stage NSCLC. Accurate nodes stage and the extent of mediatinal nodes dissection affect the peri-operative complications and the prognosis of NSCLC greatly. In the past decade, more and more surgeons demostrated that lobe-specific or selective mediastinal lymph node dissection is suitable for clinical stage Ⅰ NSCLC, especially the stage Ⅰa lesions, and may become the standard lymph node dissection mode in the future.

肺癌是我国发病率和死亡率最高的恶性肿瘤，其中非小细胞肺癌（non-small cell lung cancer, NSCLC）约占肺癌80%^[1]^，而且NSCLC已成为全球范围内癌症死亡的首要原因^[2]^。2015年，美国新诊断肺癌患者约221, 200例，约158, 040例患者死于肺癌^[3]^。在我国，无论是男性还是女性，城市或乡村，肺癌死亡率均位居癌症死亡的首位^[4]^。肺癌早期诊断、准确分期及适当的治疗方式对于提高肺癌患者生存率至关重要。随着影像学技术的飞速发展及人们健康意识的不断提高，早期肺癌，尤其是肺部膜玻璃结节（ground grass opacity, GGO）的诊断比率越来越高。2015年第7版美国国立综合癌症网络（National Comprehensive Cancer Network, NCCN）指南指出外科手术治疗仍是Ⅰ期、Ⅱ期和部分Ⅲa期NSCLC最主要的治疗方式^[5-7]^。而纵隔淋巴结转移是影响NSCLC患者预后的一个重要因素，因为早期肺癌手术治疗时完全清扫转移淋巴结对提高患者总生存率及术后无病生存率起着关键作用^[8]^。因此，术前和术中正确评估早期NSCLC患者淋巴结转移情况对于选择适当的淋巴结清扫方案起着关键作用。然而，术前很难明确判断早期NSCLC患者纵隔淋巴结受累情况，因此，临床上对早期肺癌，尤其是临床Ⅰ期NSCLC患者手术过程中纵隔淋巴结清扫方式一直以来存在很大争议。本文对国内外NSCLC淋巴结转移规律和淋巴结清扫方式的相关研究结果进行综述。

## 1 早期NSCLC的淋巴结转移规律研究进展

### 1.1 生理性肺淋巴液引流规律的研究

研究者们^[9, 10]^曾通过灌注技术从解剖学上研究肺的淋巴引流方式，然而这仅能够发现浅表的胸膜下淋巴结引流途径，却难以发现肺内淋巴结引流，同时注射速率也能影响其研究结果。组织胞浆菌病^[11]^是一种原发性真菌病，常侵犯到肺引起原发部位实变，并累及肺门及纵隔淋巴结引起相应淋巴结钙化，Takahashi及Stanford等^[12]^通过分析该病计算机断层扫描（computed tomography, CT）表现的分析发现肺部病变的淋巴液经肺门到纵隔淋巴结引流遵循一定规律，并设想该规律为肺部淋巴液在生理情况下的引流方式：右肺上叶淋巴液易引流至4R区、肺门和肺内淋巴结，右中叶淋巴液易引流至7区、4R区、肺门及肺内淋巴结，右下叶淋巴液易引流至7、9、8区、肺门及肺内淋巴结，左上叶肺淋巴液易引流至5、7、4L区、肺门及肺内淋巴结，左下肺淋巴液易引流至7、8、9区、肺门及肺内淋巴结。

### 1.2 肺癌原发部位与淋巴结引流路径的研究

NSCLC纵隔淋巴结转移与否对于患者准确的临床分期及制定相应的治疗方案至关重要，对手术清扫方式有非常重要指导意义，尤其对于早期NSCLC更为明显。近年来，国内外学者进行了大量的研究以寻求不同肺叶NSCLC的纵隔淋巴结转移规律。

希腊学者回顾性研究^[13]^557例术前诊断为淋巴结病变阴性的NSCLC且均行手术治疗加系统性纵隔淋巴结清扫的患者，其研究显示不同肺叶原发NSCLC纵隔淋巴结转移表现出一定规律：右上叶肺癌主要转移至4R区、肺门和肺内淋巴结，右肺中叶癌主要转移至7、4R区、肺门和肺内淋巴结，右肺下叶癌主要转移至7区、肺门和肺内淋巴结，左肺上叶癌主要转移至5区、肺门和肺内淋巴结，左肺下叶癌主要转移至7、8、9区、肺门和肺内淋巴结。而且相关研究^[14, 15]^关于NSCLC原发部位与淋巴结转移的关系曾有类似的报道。Cerfolio等^[16]^及其他学者^[17, 18]^对不同肺叶原发NSCLC发生淋巴结转移的规律的研究结果与以上类似，同时还发现与原发于左侧的肺癌相比，原发于右侧肺叶的肺癌更容易发生纵隔淋巴结转移（*P*=0.02）。

国内研究者^[19]^报道对于直径≤3 cm的周围型NSCLC，肿瘤直径越大，其纵隔淋巴结转移率越高，上肺叶肺癌主要转移至上纵隔淋巴结，下肺叶肺癌则隆突下及上纵隔均可转移，纵隔转移的淋巴结在左肺主要分布在第5、6、7组、肺门及肺内淋巴结，而右肺主要分布在4、7组、肺门及肺内淋巴结，并指出术中应该重点清扫以上区域淋巴结。

日本学者^[20]^1999年也报道166例伴有N2淋巴结转移患者的各肺叶发生纵隔淋巴结转移的规律，其结果显示：右肺上叶肿瘤转移性N2淋巴结主要是4R、2R区；右肺中叶转移性N2淋巴结主要位于7区，其余上下纵隔也有少数阳性转移；右肺下叶主要转移到7、8、9区，左肺上叶主要转移到5、6区，左肺下叶主要转移7、8、9区。然而这组研究结果均为伴有N2阳性的患者，不能代表早期肺癌淋巴结转移规律。

### 1.3 早期肺癌淋巴结转移规律

日本学者Okada等^[21]^通过对956例早期原发性肺癌行系统性淋巴结清扫总结早期肺癌淋巴结转移规律，总结出各肺叶病变易发生转移淋巴结区域规律如下：右上叶病变：2R、4R区；右肺中叶：2R、4R及7区；右下叶病变：7、8、9区；左上叶病变：5、6、4L区；左下叶病变：7、8、9区，并将以上区域定义为肺叶特异性引流区域。

Fujiu等^[22]^回顾性分析192例T1期NSCLC行系统性淋巴结清扫后淋巴结转移规律：69例右肺上叶患者中仅1例发生隆突下淋巴结转移（1.4%），而10、11及特异引流区（2R及4R区）阴性时，发生下纵隔淋巴结转移率为0.0%；当肺门和特异性引流区淋巴结无转移时，33例右肺下叶患者发生上纵隔淋巴结转移的概率也是0.0%；51例左肺上叶患者也均无下纵隔淋巴结转移0.0%；22例左肺下叶患者中，仅1例伴有7区淋巴结转移患者，出现上纵隔非特异性引流区淋巴结转移（4.5%），该研究也证实当特异性引流区淋巴结冰冻病理为阴性时，非特异性引流区淋巴结可不予清扫。

我院通过回顾性分析^[23]^直径≤3 cm的临床Ⅰ期行系统性淋巴结清扫术的270例NSCLC患者显示：直径≤1 cm组与1 cm < 直径≤2 cm组的淋巴结转移率差异无统计学差异（*P*=0.653），但1 cm < 直径≤2 cm组与2 cm < 直径≤3 cm组的淋巴结转移率差异有统计学意义（*P* < 0.01）。本组34例纯GGO患者无淋巴结转移（0.0%），47例混合型结节淋巴结转移率为2.1%，而189例实性结节组的淋巴结转移率分别为，26.5%，后两组与纯GGO组相比差异有统计学意义（*P* < 0.05）。我们同时发现肺门和特异性引流区阳性时，右肺上叶、右肺下叶，左肺上叶，左肺下叶非特异性引流区淋巴结转移率分别为30.0%、20.0%、44.4%和50.0%，而肺门和特异性淋巴结引流区淋巴结阴性时，右肺上叶、右肺下叶，左肺上叶，左肺下叶非特异性引流区淋巴结转移率分别为0、0、0和2.9%，差异均有统计学意义（均*P* < 0.05）。依据上述早期肺癌淋巴结转移规律，作者建议对于影像学表现为p-GGO的NSCLC，可以仅对特异淋巴引流区淋巴结取样或不行淋巴结清扫；对于≤2 cm的结节型或混合型Ⅰ期NSCLC，术中可考虑先清扫肺叶特异性淋巴引流区淋巴结送冰冻，若术中冰冻病理检查为阴性，则不行非特异性淋巴引流区淋巴结清扫；若为阳性，需进一步行系统性淋巴结清扫；而对于 > 2 cm的实性肺结节cⅠ期NSCLC患者，由于淋巴结转移比率较高，建议行系统性淋巴结清扫。

## 2 早期肺癌手术淋巴结清扫方式的研究进展

肺癌外科手术治疗失败的主要原因是术后复发和转移，淋巴结转移是肺癌转移的重要途径，也是初期肺癌外科治疗失败后的重要原因。因此，Churchil于1950年在美国胸部外科年会上首次强调了肺癌手术中纵隔淋巴结清扫的重要性^[24]^。最初被用于临床的淋巴结切除方式主要有纵隔淋巴结取样术（mediastinal lymph node sampling, LS）及系统性纵隔淋巴结清扫术（systematic mediastinal lymphademectomy, SML）。LS指将术中肉眼观察肿大或手感质硬，怀疑有转移的同侧纵隔淋巴结摘除，主张纵隔淋巴结采样的理由主要包括：创伤小，手术时间短，术后胸管引流少及缩短住院时间。但在1992年，Kerr等指出纵隔淋巴结大小与转移间无统计学意义。SML则是将纵隔淋巴结连同周围脂肪组织一并切除，Izbicki等^[25]^于1998年首次前瞻性对比系统性淋巴结清扫与纵隔淋巴结采样术，结果显示系统性纵隔淋巴结清扫术是一种安全术式，虽然对该组肺癌患者总的生存率没有显著提高，但提高了N1或单站N2肺癌患者的长期生存率，延长了无病生存期和降低了局部复发率。2001年吴一龙等^[26]^通过单中心、前瞻性、随机对照临床研究探讨Ⅰ期-Ⅲa期NSCLC纵隔淋巴结的最佳处理模式，评价指标包含1年、2年、3年、4年、5年、9年生存率、复发率、转移率，发现系统性淋巴结清扫后的Ⅰ期患者生存率明显高于淋巴结取样组，此外该研究认为依据系统性淋巴结清扫结果，患者的术后病理分期更准确，手术创伤虽有所增加但完全在患者可接受范围，因此，作者认为系统性纵隔淋巴结清扫应成为非小细胞肺癌的规范性术式，因此，基于上述观点纵膈淋巴结取样逐渐被纵隔系统性淋巴结清扫所替代。

一直以来，解剖性肺叶切除加系统性淋巴结清扫被认为是临床Ⅰ期、Ⅱ期NSCLC的标准手术方式。国内学者^[27]^研究报道直径在2 cm-3 cm间的Ⅰa期NSCLC经系统性淋巴结清除组5年生存率和无病生存率明显高于系统性淋巴结取样组，并认为早期NSCLC手术中行系统性淋巴结清扫对提高患者总生存率及无病生存率具有重要意义。Lardinois等^[29]^研究也认为对于临床Ⅰ期NSCLC进行系统性纵隔淋巴结清扫可明显提高患者术后无病生存率及减低术后局部复发率，同时该研究还认为系统性淋巴结清扫与系统性淋巴结取样相比，并不增加患者围手术期并发症发生率及住院时间。然而，ACOSOG Z0030实验组（American College of Surgery Oncology Z0030 Trial）对1, 023例诊断为T1-2N0-1期的NSCLC患者进行随机对照研究^[38]^报道：498例行纵隔淋巴结取样，5年无病生存率为68%；525例行系统性淋巴结清扫，5年无病生存率为69%。两组患者术后局部复发率及远处转移复发率无差异。因此，ASOSOG Z0030实验组专家认为对于术中系统性行肺门淋巴结及纵隔淋巴结取样未发现淋巴结转移的早期肺癌患者，系统性淋巴结清扫对改善患者生存率无统计学意义，其研究还发现两组对比局部复发率、区域复发率及远处转移复发率均无统计学意义，其*P*值分别为0.52、0.10及0.76。因此，对于早期肺癌，系统性淋巴结取样与系统性淋巴结清扫可以达到类似效果。

虽然，临床Ⅰ期肺癌总体分析结果显示系统性淋巴结清扫有益总体生存和减少局部复发。但对于早期肺癌尤其是Ⅰa期肺癌是否一定需要行系统性纵隔淋巴结清扫一直存在争议。目前临床上对系统性纵隔淋巴结清扫的长期预后作用及围手术期并发症的问题争议较大。近年来，国内外临床医生对早期NSCLC（尤其是临床Ⅰ期）手术过程中纵隔淋巴结的清扫方式进行大量研究并提出了早期肺癌肺叶特异性或选择性淋巴结清扫慨念，早期肺癌肺叶特异性或选择性纵隔淋巴结清扫是根据肺癌原发病灶部位，清扫各肺叶相应特异性淋巴引流区域淋巴结（肺门+纵隔淋巴结及其周围组织），并在手术中送快速冰冻病理检查，当特异性淋巴引流区淋巴结冰冻病理检查阴性，即终止其他纵膈区域淋巴结清扫；若特异性淋巴引流区淋巴结冰冻病理检查阳性，则需进一步行其他纵膈区域淋巴结清扫即改成系统性淋巴结清扫。这一手术方式既避免了纵隔淋巴结取样时遗漏部分隐匿性纵隔淋巴结的弊端，同时又减少了纵隔淋巴结清扫程度，因而减少围手术期并发症，同时不影响患者术后总体生存率及无病生存率。近十余年来越来越多的研究结果显示肺叶特异性或选择性淋巴结清扫具可行性和有效性^[30]^。

日本学者Okada等^[21]^通过对956例早期原发性肺癌行系统性淋巴结清扫总结早期肺癌淋巴结转移规律，并早在1998年首次提出选择性淋巴结清扫的概念：即下叶肿瘤未累及肺门及下纵隔淋巴结，则上纵隔淋巴结无需清扫；上叶肿瘤的肺门及上纵隔淋巴结术中未发现转移，则下纵隔淋巴结无需清扫。其指出依据肺原发病变部位术中冰冻证实相应肺叶间淋巴结、肺门淋巴结及各肺叶特异性淋巴引流区淋巴结阴性后，无需对其他非特异淋巴引流区纵隔淋巴结进行清扫。Okada等^[28]^提出选择性淋巴结清扫后，为证实选择性淋巴结清扫对早期NSCLC有效性，将后期收治临床Ⅰ期377例NSCLC患者前瞻性入组行选择性淋巴结清扫，与前期358例行系统性淋巴结清扫患者进行对比研究，研究结果显示两种淋巴结清扫方式在无病生存率（*P*=0.636）和总生存率（*P*=0.119）方面无明显差异；通过多因素分析提示肺叶特异性淋巴结清扫方式对无病生存率无明显影响，两组手术相关病死率、肿瘤远处转移和局部复发率无统计学差异，因此，Okada等^[28]^认为对于临床Ⅰ期NSCLC，行选择性纵隔淋巴结清扫可以达到与系统性淋巴结清扫相同的临床疗效，且更符合近10年来微创外科发展的理念，可以取代系统性淋巴结清扫成为临床Ⅰ期NSCLC的标准清扫方式。

国内学者^[31]^回顾性分析379例病理证实为T1a-2aN0M0的早肺癌患者，对比系统性淋巴结清扫与肺叶特异性淋巴结清扫显示两组总体3年及5年生存率均无统计学差异（*P* > 0.05），但在手术时间、术中失血、胸管引流量、拔管时间及住院天数等方面，两组存在明显差异（*P* < 0.01）；两组术后并发症亦有统计学差异（*P* < 0.05），认为肺叶特异性淋巴结清扫可明显减少手术并发症并降低围手术期风险。

综上所述，为减轻早期肺癌患者手术损伤及痛苦，肺叶特异性或选择性纵隔淋巴结清扫有可能取代既往的系统性纵隔淋巴结清扫，成为部分早期NSCLC（尤其是临床Ⅰ期）纵隔淋巴结清扫可选择的清扫方式。但目前还缺乏随机分组研究的有力证据，未来期待更多研究证明依据术中冰冻检查特异性淋巴引流区淋巴结（肺门+肺叶特异纵隔引流区）是否转移来决定清扫方式：当特异性淋巴引流区淋巴结转移阳性时行系统性淋巴结清扫，若为阴性则行肺叶特异性或选择性淋巴结清扫，从而有效避免临床Ⅰ期肺癌过度淋巴结清扫，并可减少围手术期并发症和保护患者术后免疫功能^[32]^。
